# Supplementary figures and images for: Generating functional protein variants with variational autoencoders
Source: PLoS Comput Biol. 2021 Feb 26;17(2):e1008736. doi: 10.1371/journal.pcbi.1008736 (PMC7946179; doi:10.1371/journal.pcbi.1008736)

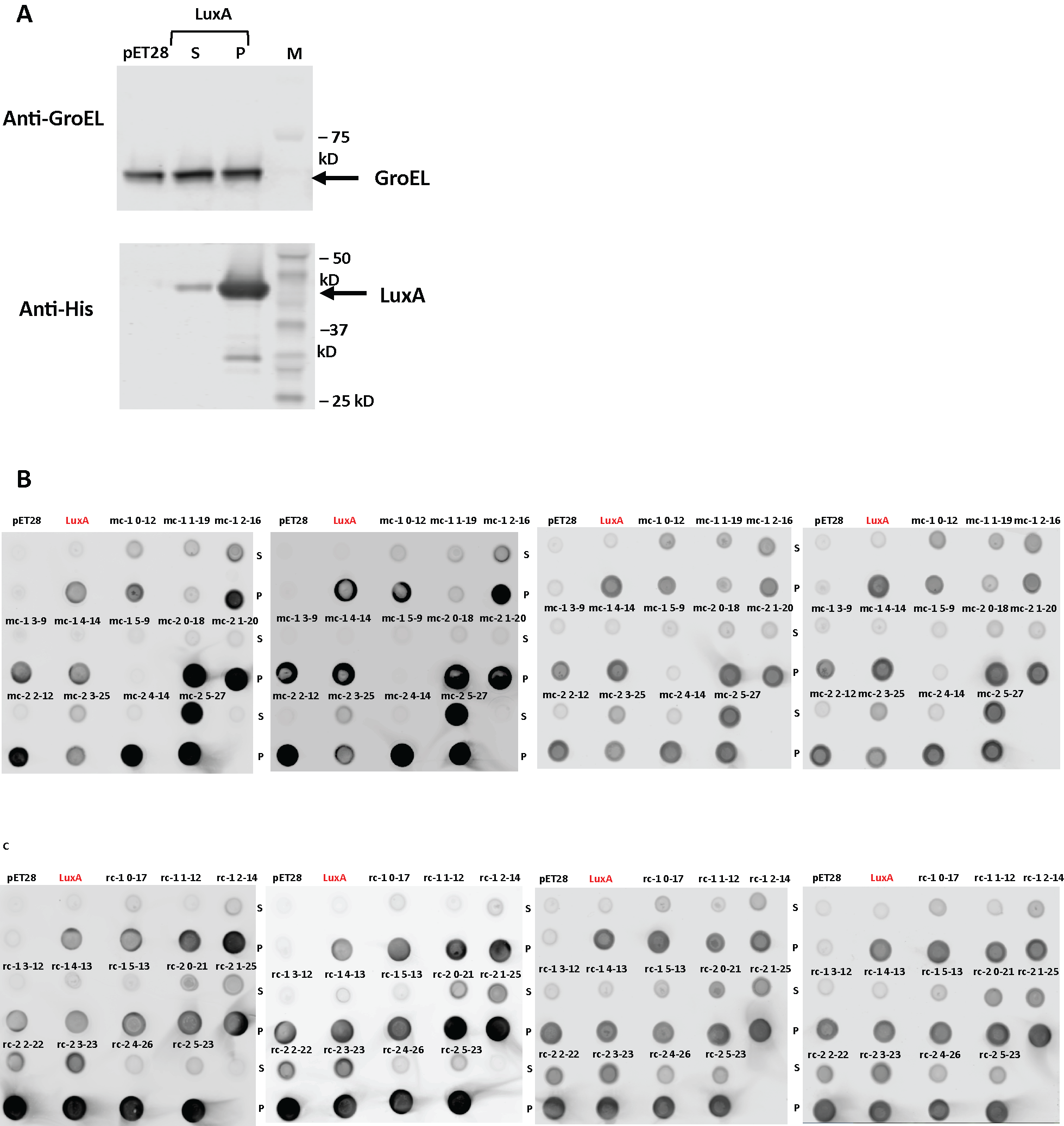

Supplement: S1 Fig — A. LuxA tagged with a His-tag was quantified by western blot in the supernatant (S, soluble fraction) and in the pellet (P, insoluble fraction) compared to the empty vector pET28. GroEL was used as loading control. The His tag was detected using the mouse monoclonal Anti-His-Tag antibody, whereas the GroEL control was detected with the mouse anti-GroEL monoclonal antibody. The molecular mass is given in kilodaltons and indicated to the right of the membrane. Arrowheads indicate the position of recombinant proteins. The levels of solubility for variants of luxA generated by models trained on aligned (B) or raw (C) sequences were analysed by dot blotting. Aliquots of 5 μl of soluble (S) and insoluble (P) fractions from IPTG-induced Rosetta cells overexpressing variants of luxA were spotted on nitrocellulose membrane and their intensities were quantified using the Image Studio software package. The dot blots of 4 technical replicates used to compute solubility are shown. (TIF) [file pcbi.1008736.s001.tif]

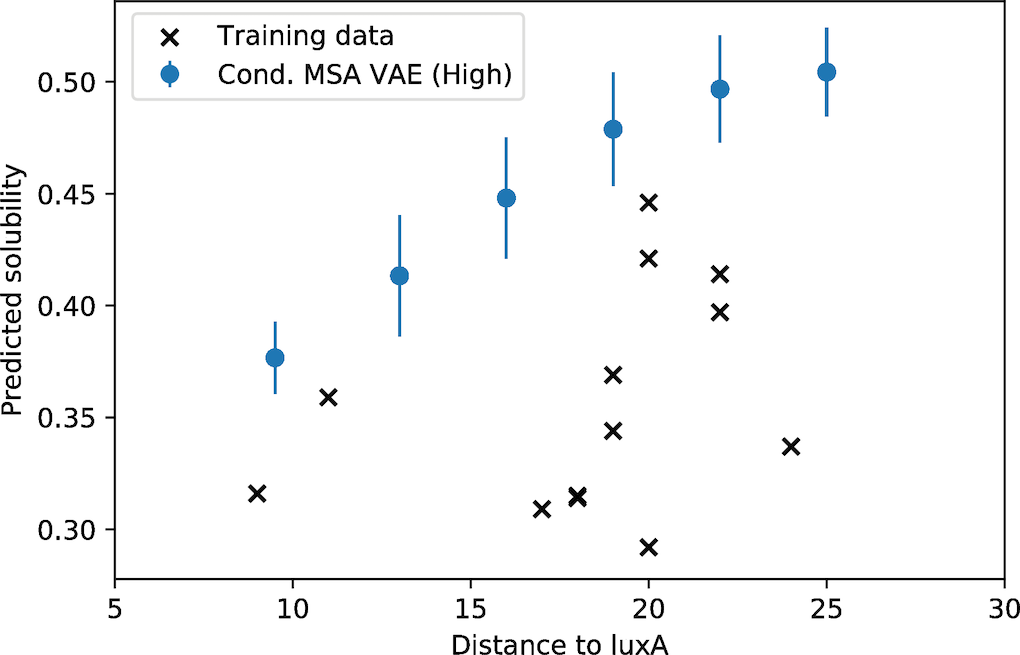

Supplement: S2 Fig — Mean and standard deviation of predicted solubility for 500 sequences generated by the conditional MSA VAE at the highest conditioning level, binned by distance to P19839 luxA, together with predicted solubility for all training sequences at equivalent distances (black crosses). (TIF) [file pcbi.1008736.s002.tif]

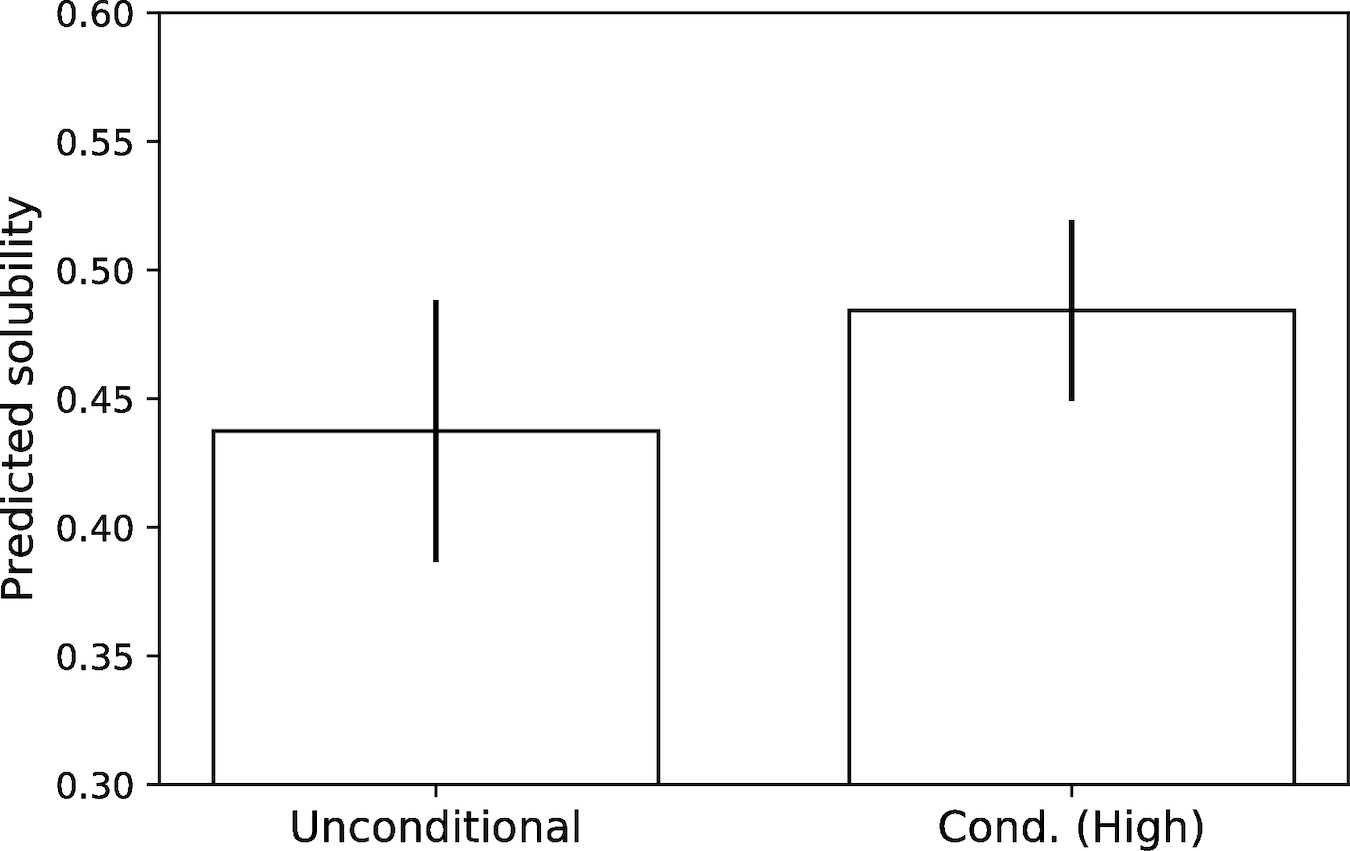

Supplement: S3 Fig — To compare conditional and unconditional models, we retrained 10 initialisations of both versions of the MSA VAE model using different random seeds. 500 variants were sampled from the posterior of the unconditional MSA VAE models and the highest level of the conditional models. For each random seed and each model, we computed the predicted solubility values of all sequences within 30 amino acid differences to luxA P19839. As a measure of the ability of the models to generate diverse high-solubility variants, we show the mean of the 50th highest predicted value across seeds. Error bars represent standard deviations. Three initialisations of the conditional model and three initialisations of the unconditional model generated insufficient variants within the distance threshold and were therefore excluded. (TIF) [file pcbi.1008736.s003.tif]

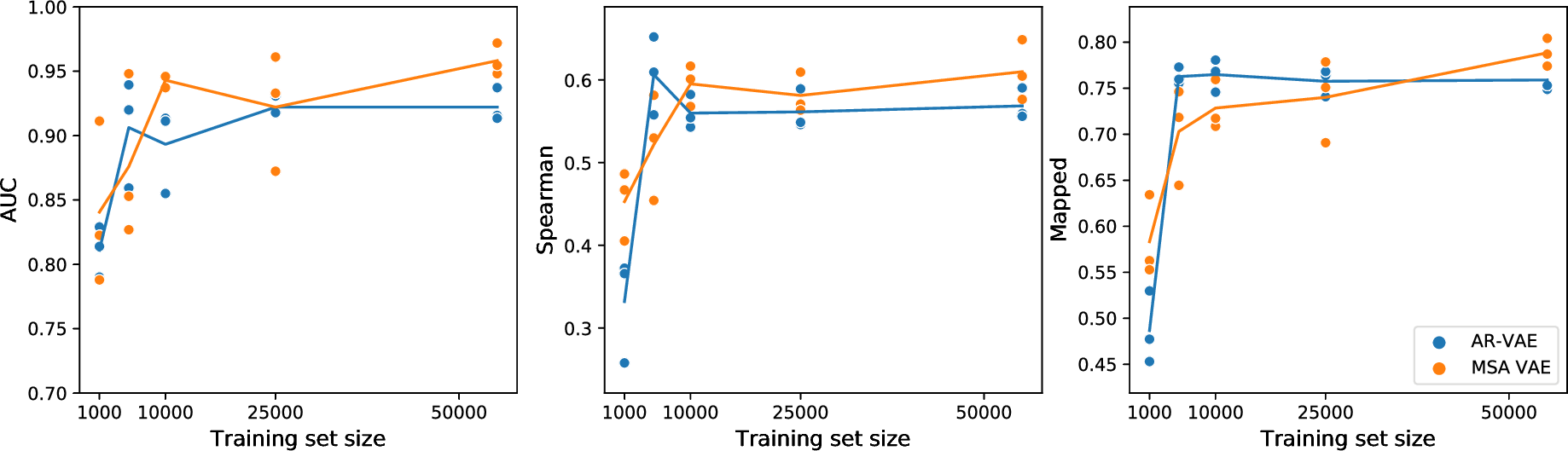

Supplement: S4 Fig — To assess the dependence of the models’ ability to learn functional constraints from naturally occurring sequences on the number of training sequences, we created reduced training sets by randomly subsampling the full training set. For each reduced dataset size, three different random subsets of the training set of that size were sampled and used to retrain models. The luxA WT sequence was included in all training sets. After training, models were assessed by their ability to predict the luminescence of the synthesised variants, using the same metrics as in Table 1. (TIF) [file pcbi.1008736.s004.tif]

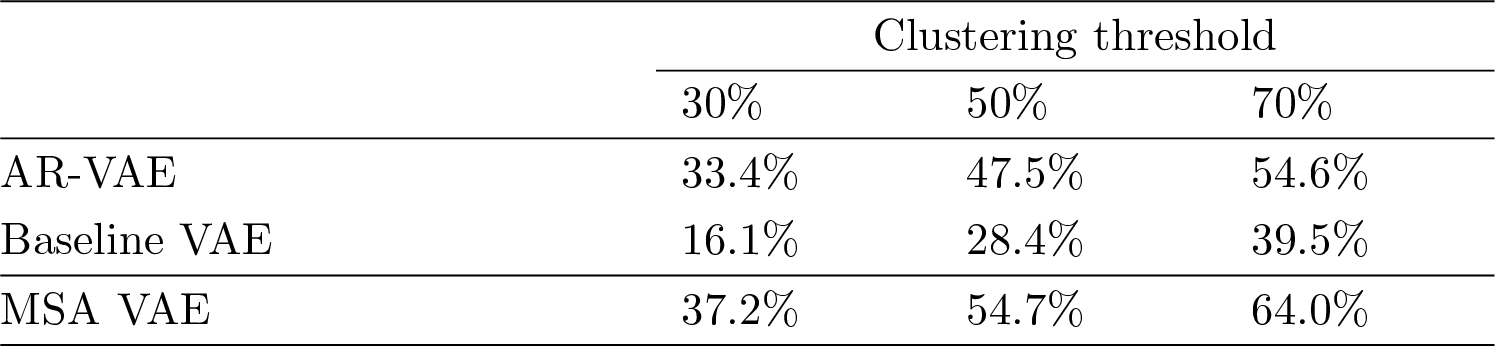

Supplement: S1 Table — To test generalisation to more distant family members, we retrained models using three different train/test splits, each of which was constructed by holding out clusters at a particular sequence identity threshold. In each case clusters were randomly added to the holdout set until the number of sequences in the holdout set was 20% of the total. Baseline VAE is a baseline model with the same architecture as MSA VAE, but the same latent dimension as AR-VAE, trained on raw sequence data. (TIF) [file pcbi.1008736.s009.tif]
