# Supplementary material for: Generating functional protein variants with variational autoencoders
Source: PLoS Comput Biol. 2021 Feb 26;17(2):e1008736. doi: 10.1371/journal.pcbi.1008736 (PMC7946179; doi:10.1371/journal.pcbi.1008736)
Supplement: Attachment — Submitted filename: 105309_1_rebuttal_1816067_qdx1d0.docx [file pcbi.1008736.s010.docx]

We appreciate the constructive comments of the reviewers, which include several suggestions that we hope will help to improve the work. Below we provide a point-by-point plan for addressing their comments in a revised version of the manuscript, and in several cases include results of the additional analysis that we intend to include in a revised submission. First we will try to respond to some of the more general comments, and reflect on our contribution in the light of them.

We openly acknowledge that use of deep generative models and other machine learning methods for the design of protein sequences is receiving growing interest, and the reviewers rightly note that such an application of these methods is not in itself novel. However, as noted by reviewer #2, “prior work has largely been restricted to computational validation”, with little attempt to convincingly assess the capacity of models to generate functional sequences. In this manuscript, by addressing a well-defined design problem and testing our success experimentally, we provide a demonstration of the practical viability of such approaches, including the use of conditioning to generate sequences with desirable properties. Together with experimental validation of a small set of sequences generated by each of four classes of model, we seek to compare the models’ performance through computational analysis of the generated sequences. We believe, with reviewer #4, that “[such] thorough assessments of generated sequences...are valuable”.

Moreover, while previous work exploring the use of deep generative models for design has focussed on training models using unaligned sequence data, we seek to draw attention to the importance of the choice of input representation for such models. In particular, we demonstrate the possibility of exploiting the rich evolutionary signal contained in multiple sequence alignments for the design of full, novel sequences by training generative models on MSAs (building on the successes of generative models trained on MSAs in tasks such as contact prediction and prediction of the fitness effects of mutations) and provide an explicit comparison of the generative capacities of separate models trained on unaligned and aligned sequences.

In terms of specific improvements we acknowledge the suggestions of reviewers that more could be done to contextualise the reported results, both by assessing the extent to which they are a function of the particular design task (and dataset) chosen, and by drawing further comparison with the kinds of results that would be expected using simpler models. In the first case, while maintaining that success in an individual design task is in itself of interest, we propose to adopt the suggestion of reviewer #1 to assess the sensitivity of model performance to the constitution and size of the training dataset by retraining models using smaller subsets and repeating the computational analysis.

In the second case, we will provide further analysis to distinguish our models from baselines, which we outline below. At the same time, we emphasise that existing understanding of protein fitness landscapes would suggest that consistently preserving function while introducing more than a handful of mutations, as done here, is non-trivial. Both experimental evidence and theoretical considerations have established that the accrual of random mutations quickly destroys protein function (Bloom et al., PNAS, 2005), and in a recent high-throughput investigation of the fitness landscape of GFP, Sarkisyan et al. showed that half of the derivatives with four mutations were completely non-fluorescent (Sarkisyan et al., Nature, 2016).

To demonstrate the differences between our models and other simple strategies mentioned by reviewers such as use of the BLOSUM matrix or profile models to identify candidate substitutions, we will provide analysis of the mutations present in sequences generated by the models, as requested by reviewer 2. Such analysis (preliminary results of which are included in the replies to the reviewers below) makes clear that these mutations differ markedly from those that would be obtained if sampling from the PFAM HMM or selecting substitutions based on BLOSUM scores. While many of the mutations are individually observed in closely related training set sequences, the success of the models in generating functional variants with tens of differences to any training set sequence suggests that the models are able to combine such (evolutionarily accessible) substitutions in novel function-preserving ways. Again, we argue that previous experimental evidence indicates that this is a non-trivial task: in the GFP study of Sarkisyan et al. it was found that while “mutations with a deleterious impact were less likely to be found in orthologous sequences...a substantial fraction of genotypes containing only mutations leading to amino acid states from GFP orthologues was non-fluorescent”. Indeed, although the vast majority of sequences containing a single substitution to another amino acid observed in an MSA of GFP orthologs tested were found to retain fluorescence, this decreased to 64% (48/75) of sequences containing 6 such mutations, and only 53% (9/17) of those containing 7 such mutations.In contrast the vast majority of sequences generated by our MSA-VAE with 20 mutations or more were functional.

Finally, we note that the experimental data we have already generated can itself be used to draw a comparison between the VAE models and simpler baselines. Interpreting the (approximate) likelihoods assigned by our models to individual sequences as continuous fitness scores, we can assess the extent to which higher fitness scores are associated with higher luminescence values in the experimental sequences via correlation metrics. We can compare these correlations to those achieved by fitness scores derived from baseline models in a similar way. The VAE likelihoods from both models (as approximated by the ELBO) show higher Spearman correlations with the observed luminescences than either HMM scores or BLOSUM scores across three different subsets of the experimental data: all experimentally tested sequences, only those generated by the MSA VAE, and only those generated by the AR-VAE. Although these results involve only a relatively small sample of sequences, they tally with more extensive prior explorations of the quality of unsupervised fitness predictions derived from generative models, which has established the superiority of VAEs to simpler baselines such as profile models and BLOSUM scores for this purpose (Riesselman et al, Nature Methods 2018).

------------------------------------------------------------------------------

Reviewer #1 (Evidence, reproducibility and clarity (Required)):

This manuscript presents an approach for the generation of functional variants of the bacterial luciferase protein, luxA. The authors develop two models; a variational autoencoder (VAE) that takes in multiple sequence alignments (MSAs) as inputs, and a VAE with an autoregressive component that takes in raw sequences as input. The authors generate sequences using both approaches and demonstrate the capacity of the model to recapitulate low-order statistical information about the family of proteins. A subset of sequences was furthermore cloned and tested for functional luciferase activity. Finally, the authors explore the incorporation of sequence solubility information into the model by conditioning the latent representation posterior distribution on predicted sequence solubility. Generated sequences were assessed for their solubility by cloning with a His-tag and performing western blots in different fractions of a protein solution.

**Major comments:**

1.The distance measurement used to demonstrate that generated sequences showed variability from the training dataset was the number of substitutions, including indels, from the sequences in the training dataset. Presumably, the generated sequences would all have a length of 360 amino acids, corresponding to the length of P19389. Since columns in the MSA where P19389 is assigned gaps were dropped, the generated sequences are guaranteed to have indels with respect to a portion of the training dataset. If the score used only considered the 360 relevant columns, it is possible that the observed distance will be less than what was reported.

We will clarify the way in which distances to the training set sequences were calculated, since it is certainly true that in general different results may be obtained if considering full sequences or if considering only the retained columns in the training MSA. The distances in question were in fact calculated using only the columns in the training MSA, as the reviewer suggests. For these specific sequences, the smallest distance to any training set sequence is almost always equivalent computed in either fashion, since the closest training set sequence will be luxA or one of its close relatives in the training set, and will contain few, if any, indels relative to luxA.

Without providing alignments, it is furthermore unclear whether the substitutions between the generated sequences and the training dataset are commonly observed in the training set and whether similar results could be achieved using a model that only makes use of low-order statistics.

Though many of the substitutions, when considered individually, are observed in related sequences in the dataset, the models’ ability to generate variants with many differences to luxA which retain function indicates that it is able to combine such substitutions in novel function-preserving ways.

2.It is not clear in Figure 5 that conditioning by solubility results in improved solubility of sequences generated by the MSA-VAE model. The baseline to which the generated sequences were compared is the wild type luciferase while a more appropriate comparison may be to sequences generated by the model without conditioning on solubility. One of the reasons the authors advocate for the use of a VAE is the ability to condition the posterior distribution for the latent representation on additional information. There is no evidence that a similar result couldn't be achieved simply by filtering generated sequences by the predicted solubility using protein-sol. This approach could be applied to any generative model.

An advantage of conditioning on solubility in the MSA VAE model that is not clear from figure 5 is that it enables us to control the solubility level while introducing relatively few mutations, indicating that including solubility as conditioning information to a certain extent disentangles solubility from other sequence features required to reconstruct a protein accurately. This is evidenced in the figure below, which highlights the fact that the generated sequences have consistently higher predicted solubility levels not only than the luxA wild type, but also than any sequence at an equivalent distance in the training dataset.

The below figure only shows the distance distribution for the highest solubility level; sampling sequences at lower solubility levels leads to sequences with fewer differences on average to luxA. In this way, conditioning on solubility balances faithful reconstruction of luxA with the introduction of sufficient mutations to satisfy the desired condition. There is no equivalent mechanism in unconditional models, and sampling from the posterior of a model able to reconstruct luxA equally as well as the conditional model will not generate sufficiently diverse sequences for filtering to be effective. Though there may be more sophisticated sampling-based approaches that achieve similar end results, we argue that this does not invalidate the use of conditioning to achieve the kind of behaviour we sought to demonstrate. We will seek to clarify these considerations in our presentation of the conditioning results

Figure: Mean and standard deviation of predicted solubility for 500 sequences generated by MSA VAE at the highest conditioning level, grouped into bins by distance to luxA, together with predicted solubility for all training sequences at equivalent distances (black crosses).

3.Applicability of the model is contingent on the ability to create a large dataset of homologous sequences. The model's performance on smaller subsets of luciferase sequences or on sequences that are not as closely related is not explored. Re-training the model on portions of the database and performing the same computational analysis can give an insight into the broader model applicability.

We agree that it would be useful to retrain the model "on portions of the database and [perform] the same computational analysis". We hope that doing this would go some way to addressing the concerns of other reviewers about how strongly the success of our approach depended on particular details of the chosen task and provide “an insight into the broader model applicability”.

4.It's not clear how good the luminescence results actually are, as there is no "negative baseline" given. While having about 3/4 of the sequences giving some luminescence seemed quite good (though all of them had reduced luminescence compared to wt, at best it appears to be roughly 1/2 or so of the wt (difficult to tell given the log10 plot)), it's not clear whether if one would, for instance, simply generated sequences from the Pfam HMM (or some other simple way), how often one would succeed at getting functional sequences. Certainly, one could imagine making ~25 (seems roughly the average their chosen sequences are away from the closest one in the alignment) mutations of the L to I type in poorly conserved positions to a 360 residue protein and expect its function to at least not be vastly diminished.

As argued in the main response above: existing knowledge of the fitness landscapes of proteins clearly suggests that introducing more than a handful of mutations while consistently preserving function is a nontrivial task. Fitness has been observed to decline exponentially with an increasing number of random mutations in mutagenesis studies (Bloom et al., Sarkisyan et al.), and Sarkisyan et al. also found that GFP sequences differing from avGFP only by substitutions which had been individually observed in close orthologues, though more likely to be functional than randomly mutated sequences with equal numbers of mutations, nonetheless also tended to lose function at much shorter distances to the wild type than we explore here (around one third of sequences containing as few as 6 mutations all observed in GFP orthologues were non-functional).

Nonetheless we agree that further comparison with simpler baseline models would strengthen our work and help in interpreting the success of the VAE models. As outlined above, we propose including two additional forms of analysis to help in this regard.

First, examining the substitutions that are favoured in the sequences generated by the VAE models shows that they differ significantly from substitutions that would be expected if using the BLOSUM matrix or the PFAM HMM to guide the generation of variants.

Second, correlating the VAE ELBOs (which are approximations to the likelihood) assigned to the synthesised sequences with the luminescence measurements for those sequences provides a means of assessing the extent to which the models assign higher probability to functional sequences. Although the numbers of sequences involved are relatively small, this analysis offers evidence that the MSA VAE likelihood in particular seems to effectively distinguish functional from non-functional variants, and to do so better than BLOSUM or HMM baselines.

Preliminary versions of these analyses are included here:

Figure: frequency of substitutions in a set of 500 sequences generated by the unconditional MSA VAE model compared to corresponding BLOSUM scores, PFAM HMM emission probabilities, and substitution frequencies in the cluster of training set sequences containing luxA. Clusters were found as explained in the main text using mmseqs2 with a clustering sequence identity threshold of 70%.

Table: Spearman correlation of luminescence values for experimentally tested sequences with each of four scores: a BLOSUM score computed by summing the scores for each of the substitutions, an HMM score computed by scoring the sequences with the PFAM HMM for the luciferase-like family, and ELBOs for the two VAE models. Sequences generated by the AR-VAE were aligned to P. Luminescens luxA before being scored by the MSA VAE. Correlations were computed for each of three subsets: the set of all sequences generated by the MSA VAE (conditional and unconditional), the set of all sequences generated by the AR-VAE (conditional and unconditional), and the set of all sequences tested, which includes the luxA wild type.

**Minor comments:**

1.The authors stated that a 70% clustering threshold was used to split the training and validation set since the "ultimate goal was the generation of variants with reasonably close similarity to the target protein". Given that clusters are assigned to the validation dataset randomly, increasing the clustering threshold is not going to necessarily result in a training dataset with higher similarity to the target sequence.

Our goal was to have a validation set that enabled us to assess generalisation of models to sequences reasonably close to the training set, since this was the setting in which we ultimately wanted to use our model (to generate luxA variants). We did not seek to construct an optimal training set for the generation of luxA itself, and agree that performance could depend on the precise way in which training and validation sequences are allocated. We will consider whether this sentence ought to be updated so as not to give a misleading impression of what we intended. This is also a question that could be explored when we select subsets of the training set to re-train on (c.f. response to major comment 3 above)

2.In Figure 2, visualization of the generated latent vectors was only shown for the MSA-VAE model. This analysis should also be shown for the AR-VAE to substantiate the claim that the projected vectors also segregate by InterPro sub-families.

We will add further support for these claims: aside from the visualisation (which we show for validation sequences for both models below), logistic regression models are able to classify latent vectors into protein families with high accuracy for both models.

Figure: PCA representation of latent vectors for validation set sequences for MSA VAE (left) and AR-VAE (right).

3.The luxA structure used to generate the contact map in Figure 4 was not indicated.

We will update the methods section to clarify the luxA structures used for contact map visualisation, which was obtained using EVCouplings, based on aggregated distance information from the pdb structures 1luc, 3fgc and 1brl. The aggregated distances are the minima of the inter-residue distances extracted from the individual structures.

4.There is a notable gap in the predicted position couplings using the sequences generated by the MSA-VAE (from positions 220 to 300), in Figure 4. This was not addressed by the authors and may represent an artifact in the multiple sequence alignment.

The columns identified contain a larger than average number of gaps; this in turn has an impact on the distribution of CCMPred scores.

Further analysis shows that there is still, however, meaningful covariation signal in this region: contacts can successfully be predicted from the training MSA in this region.

5.It's not clear what is going on with the "HMM sequences" used for Fig 3. Were these sequences just from sampling the PWM (generated from the Pfam alignment) or were they generated using the actual HMM? In either case, the third plot with some coupling signals zero and the others essentially correlating well with the "real" coupling doesn't make sense.

This helpfully points out a confusing feature of Figure 3. We had included in the plot entries from the diagonal of the covariance matrix, where entries correspond to variances of amino acids at individual residues rather than covariances between amino acids at different residues. Excluding these gives a much clearer picture, which we will include in our revised submission.

The sequences were sampled from the PFAM HMM, but statistics were only computed at match states. This is equivalent to sampling from the HMM without allowing insertions, which in turn is (almost) equivalent to sampling from the emission probabilities at each match state position of the profile HMM.

6.A portion of the sequences generated without conditioning on the solubility of the seed sequences showed no luciferase activity while all the sequences generated while conditioned on solubility showed luciferase activity. Presumably the latter sequences were biased by the fact that the authors needed to detect their expression with the anti-HisTag antibody, while there is no data showing that the sequences lacking luciferase activity were actually expressed. Reporting the aggregate numbers from both experiments together in the abstract is thus somewhat misleading.

There was no bias in the sequence selected for the solubility measurements in the sense that we did not select well expressed proteins in this assay. The fact that we could detect luminescence for all the variants tested in this assay is likely due the fact that these sequences are closer to the WT LuxA protein than variants generated without conditioning.

Reviewer #1 (Significance (Required)):

There has been a fair bit of work now on using various generative methods for protein sequence information (a subset of which has been cited here). I see this particular work as among the less ambitious ones, applying VAEs to a well-defined and not hugely difficult problem. There is some success reported, though I find it difficult to ascertain how impressive it is.

As argued in the main response: although other work with generative models has been proposed, we draw attention to the choice of input representation and the benefits of using alignments where available, in contrast to previous papers exploring design with deep generative models that we are aware of. We also differ from much previous generative modelling work in demonstrating the use of generative models to tackle a well-defined and non-trivial design goal, and verifying success experimentally. We believe that attempting to make such approaches practical itself contains important lessons for further developments of these methods.

Reviewer #2 (Evidence, reproducibility and clarity (Required)):

**Summary**

The work aims to generate modified amino acid sequences that are variants of naturally occurring proteins while retaining the wildtype's function and optionally increasing its solubility. For this purpose the work proposes the use of variational autoencoders to learn a distribution over the sequences of a given protein's family, which can additionally be conditioned on the sequences' in silico solubility score. This framework is applied to one scenario, the generation of luxA variants. It is shown experimentally that a selected set of generated sequences for luxA retains similar bioluminescence as the wildtype and that additionally it is possible to increase solubility of the wildtype by appropriately conditioning the generative models.

Overall the work makes for an interesting read and is presented in a clear and well structured fashion. Aside from experimental validation of a small set of generated sequences, the work offers interpretations of how the generative models represent the sequences and carries out statistical analyses into how well the models capture the training set statistics on both the single and pair amino acid level.

**Major Comments**

I have two main comments:

For one, the similarity of the generated sequences to the training set is high (as acknowledged), but there seems to be no analysis into whether the substitutions the model makes are trivial. For example, it would be important to analyse whether the substitutions are simply such that are permissible under the Blosum62 matrix. Similarly the model is trained on a dataset with a 70% sequence identity threshold, what is the median sequence similarity here though and can they be interconverted by using Blosum62 substitutions? Another important analysis would be to use control sequences in both the bioluminescence and solubility experiments, e.g. what is the luminescence (and solubility) of the wildtype sequence with up to 30 substitutions using i) Blosum62 substitutions, ii) the HMM model and iii) random substitutions? This would be important to know as context for the model generated sequences.

We agree that we could do more to interpret the kinds of substitutions made by the models when generating luxA variants. One way of starting to explore this is by comparing the frequencies with which substitutions are made by the models to the frequencies with which they occur in the training set, and with their probabilities/scores under the BLOSUM and PFAM HMM models.

Preliminary analysis along these lines for the MSA VAE model is shown below, and shows that the frequency of substitutions shows than neither the BLOSUM scores nor the PFAM HMM probabilities correlate well with the frequency of substitutions. We will seek to extend this analysis and add our conclusions to the manuscript.

Figure: frequency of substitutions in a set of 500 sequences generated by the unconditional MSA VAE model compared to corresponding BLOSUM scores, PFAM HMM emission probabilities, and substitution frequencies in the cluster of training set sequences containing luxA. Clusters were found as explained in the main text using mmseqs2 with a clustering sequence identity threshold of 70%.

In terms of the suggested baselines, as argued in the main response above: existing knowledge of the fitness landscapes of proteins clearly suggests that introducing more than a handful of mutations while consistently preserving function is a nontrivial task. Fitness has been observed to decline exponentially with an increasing number of random mutations in mutagenesis studies (Bloom et al., PNAS, 2005), and in a recent high-throughput investigation of the fitness landscape of GFP, Sarkisyan et al. showed that half of the derivatives with four mutations were completely non-fluorescent (Sarkisyan et al., Nature, 2016). Sarkisyan et al. also found that GFP sequences differing from avGFP only by substitutions which had been individually observed in close orthologues, though more likely to be functional than randomly mutated sequences with equal numbers of mutations, nonetheless also tended to lose function at much shorter distances to the wild type than we explore here (around one third of sequences containing as few as 6 mutations all observed in GFP orthologues were non-functional).

To add further support to our claim that our models outperform simple baselines we propose to examine the correlation of our models’ likelihoods with the experimentally determined luminescence values, as outlined in the main response. This analysis offers evidence that the MSA VAE likelihood in particular seems to effectively distinguish functional from non-functional variants, and to do so better than BLOSUM or HMM baselines.

Table: Spearman correlation of luminescence values for experimentally tested sequences with each of four scores: a BLOSUM score computed by summing the scores for each of the substitutions, an HMM score computed by scoring the sequences with the PFAM HMM for the luciferase-like family, and ELBOs for the two VAE models. Sequences generated by the AR-VAE were aligned to P. Luminescens luxA before being scored by the MSA VAE. Correlations were computed for each of three subsets: the set of all sequences generated by the MSA VAE (conditional and unconditional), the set of all sequences generated by the AR-VAE (conditional and unconditional), and the set of all sequences tested, which includes the luxA wild type.

For another I don't really see the necessity for a generative model to operate on sets of unaligned sequences, and do not find it very surprising that this works worse than operating on aligned sequences in the presented setup. If I understand correctly, each instance in the training batch is a set of sequences. In the case of the AR-VAE these are unaligned and therefore the model's encoder is impeded in finding a compressed latent representation based on using co-variances in the sequences. The autoregressive model on top seems to largely ignore long-range interactions and in combination this results in an inferior performance compared to the MSA-VAE. The question is why one would want to learn the distribution of unaligned sequences as in the AR-VAE in the first place. To circumvent alignment, it is not necessary to present sets of unaligned sequences as batch instances, instead one can operate on single sequences as batch instances (as e.g. in [1]).

If we understand correctly, one of the reviewer's questions is why we take a latent-variable based generative modelling approach when working with unaligned sequences rather than the purely autoregressive approach used in e.g. ProGen. We agree that this is an important aspect of our choice of design and will try to update the relevant sections of the manuscript to further clarify this. Our reasoning is that a latent variable model offers various potential advantages over a purely autoregressive model: (i) greater diversity of samples (c.f. e.g. Bowman et al, 2017), (ii) the related fact that there is no need to condition the generation with an initial seed sequence (corresponding to e.g. the first section of the protein, which would only allow the model to propose variations at a subset of positions) and (iii) the possibilities that latent space affords for control of generated sequences e.g. via searching for sequences with desirable properties (Gomez-Bombarelli et al. 2018) or other operations on the latent codes.

Given the choice of a latent variable based modelling approach, the use of an autoregressive decoder helps to overcome one of the problems identified in the prior latent variable modelling work for unaligned sequences identified by the reviewer (i.e. Greener et al.), where a non-autoregressive model similar in architecture to our MSA VAE here was found to be unable to reproduce the second-order statistics: in their discussion, Greener et al. note: ‘Sequences generated by the model described here show similar conservation patterns to native sequence families. However, the covariation of residues present across sequence families is not reproduced by the model.’ Indeed we offered explicit comparison with the purely feed-forward architecture in the results and in Supplementary Table 1. Our model thus addresses one of the weaknesses of this previous latent variable model of unaligned sequences, although we acknowledge limitations in the approach too, particularly with regard to long-range interactions as noted by the reviewer and discussed in the text.

As for the question of batch construction, our approach is standard (for VAEs trained on sequence data): training is performed using gradients of the negative ELBO loss computed over minibatches. Each item of the minibatch is a single sequence, which is passed through the encoder to obtain a latent vector, from which a reconstruction is decoded. The minibatch loss is the ELBO evaluated across all sequences in the mini batch. We hope that this clarification answers the reviewer’s concern and will revise any misleading sections in the manuscript.

**Minor Comments**

Inferred contact maps (Fig. 4):

I find them a bit unconvincing.

The work finds 'reasonable resemblence' between the contact maps. Could this be quantified, e.g. in terms of a likelihood or cross-entropy?

We wanted to emphasise the qualitative character of the analysis: the contact maps provide a visually interpretable picture of the dependencies captured by the models, that helps in the comparison of AR-VAE and MSA VAE models.

ML models:

The number of used parameters of the two VAEs seems to differ quite a lot (AR-VAE is far deeper). Why is that?

Why did the MSA VAE not get a convolutional decoder like the AR-VAE (and perhaps a convolutional encoder)?

Why is the encoder distribution artificially broadened? Was this informed by experiments?

The differences in architecture between AR-VAE and MSA VAE are discussed briefly in the text, but we agree that our discussion could be expanded, as it currently focusses on the decoders. Working with an MSA rather than unaligned sequences diminishes the need to model local sequential dependencies. Fully-connected models, unlike standard sequential models, do not have any bias towards local dependencies, and are therefore a natural choice for capturing arbitrary dependencies between residues in MSAs. They are also conceptually simpler and faster to train, while empirically we found no advantage to using sequential models.

Interpretability of ML models:

I miss a discussion of the somewhat random nature of the structure of the latent / representation space. E.g. how does this change across training runs? This structure usually emerges somewhat stochastically as the training process is stochastic, which should perhaps be discussed.

We will seek to address this in the text.

Solubility:

Discuss protein-sol? It appears it is sequence based only, discuss the limitations of this approach as structure based tools seem to be more accurate? Also, in this work, it appears that an overall negative charge for the protein is beneficial for solubility. Usually the most important features seem to be the presence of hydrophobic amino acids on the structure's surface as well as an alternating pattern of charged residues on the surface?

The solubility experiments show a somewhat poor correlation between the employed conditioning tag and solubility, could this be limited because of protein-sol's limitations?

Protein-sol indeed relies on sequence features only. Such an approach seems more appropriate for a case like ours where we are modelling the distribution of sequences for a set of proteins with similar structure. In particular, since we lack detailed structural information for the majority of individual proteins in our training dataset, a structure-based solubility predictor would have no basis on which to generate protein-specific solubility predictions. Nonetheless limitations of protein-sol (and solubility prediction in general) naturally constrain the success and degree of control which can be expected when conditioning on predicted values.

Negative overall charge is one of the most important sequence-based solubility-promoting features not only in protein-sol but also in other solubility prediction methods (Hou et al., 2020), explaining its prominence in our analysis.

We will seek to clarify these issues in the text by extending our discussion of the ways in which the models promote solubility, both in the context of the capacities and limitations of protein-sol and in terms of the broader understanding of the basis of solubility.

Missing references:

Line 55: Stochastic backpropagation and approximate inference in deep generative models (Rezende et al.).

Line 66: Generative models for graph-based protein design (Ingraham et al.), ProGen: Language Modeling for Protein Generation (Madani et al.).

Line 79: Generative models for graph-based protein design (Ingraham et al.), ProGen: Language Modeling for Protein Generation (Madani et al.).

We apologise for missing these references and will add them in our revised submission.

Reviewer #2 (Significance (Required)):

Compare to existing published work

Variational autoencoders and autoregressive generative models have been used for the generation of novel protein sequences before. Both for unconditional and conditional sequence generation [1, 2]. I am however not aware of work that specifically conditioned on (and optimized for) solubility and it was good to see that there was experimental validation of a set of the generated sequences, as prior work has largely been restricted to computational validation.

Audience

The appropriate audience for this work appears to be computational biologists and machine learning practitioners working on the design of protein sequences.

Your expertise

My expertise lies in generative machine learning models, protein structure prediction and design.

References

[1] ProGen: Language Modeling for Protein Generation (Madani et al.)

[2] Design of metalloproteins and novel protein folds using variational autoencoders (Greener et al.)

Reviewer #3 (Evidence, reproducibility and clarity (Required)):

How do these results compare to other efforts to design GFP variants or other protein classes? This is, on some levels a protein design paper, protein design is a mature field that needs be discussed in greater depth if the reader is expected to make sense of your empirical results and their overall significance.

We have briefly sketched existing design strategies in the introduction, but agree that we could do more to situate our approach with respect to them, and will extend this aspect of the discussion in our revised submission.

Performance on alignment is great, performance on contact prediction is ok (but not great). Would it make sense to focus in greater depth on one of the downstream bioinformatics applications you can explore, instead of a tour of many?

We provide the analyses of alignment statistics and inferred contacts to help assess the properties of the generated sequences (and the models that generate them), rather than to provide examples of downstream applications. As such we do not seek to demonstrate state of the art performance on contact prediction, and are mainly interested in what the analyses tell us about the generative capacities of the models and their suitability for design tasks.

Can you generate new things? A problem with VAEs is that if you want to generate a new cat you only train on cats. That is, if you want something completely different than luciferase-like oxidoreductases (e.g., a protein sequences with some other function) using their approach, you'll have to start everything from scratch (collect sequences with properties/functions you are interested in, run MSA, train VAE again, sample new sequences, etc, etc...). Their conditional VAE doesn't really explore new data in order to predict proteins with novel functions. Are you constrained with the number of training sequences (with the desired properties) you can provide?

The models are limited to the domain on which they are trained; but this is not particular to our approach. As we mention in the discussion, models which work with input from MSAs can only learn the distribution of sequences which can be represented within the MSA: therefore the properties and functions of the sequences that are generated by the model are naturally expected to be similar to those of the sequences in the training alignment (but the actual sequences may still differ substantially from any training set sequence: the ability to generate novel proteins with similar function to training set sequences is precisely what we seek to demonstrate).

Considering just the problem of generating new variants of a protein with a given function, we agree that the extent to which the precise constitution and size of the training dataset determines performance is worthy of further exploration, and have outlined above our plan to address this.

In other situations it might be desirable to have the possibility of generating sequences with a wider diversity of functions; this would require training a model on a wider range of input proteins (e.g. from multiple families). The MSA VAE cannot be straightforwardly extended to this setting, but the AR-VAE could, since it can work with unaligned sequences and therefore imposes no restrictions on the relationships between training sequences. We agree that such considerations are of interest, but think that further exploration of these issues is beyond the scope of this work.

The decoder described is autoregressive, which means the sampling of sequences (in the post training phase) is very slow (residue by residue hoping to hit a stop symbol to generate a sequence of different length than the lengths of training sequences).

Is there a faster non-autoregressive method possible, one that could be sampled faster?

While it is true that sampling from the AR-VAE is slow it is not prohibitively so for our application. We nonetheless agree that exploration of alternative models which permitted faster sampling would be an interesting avenue for future work, particularly in applications where a large number of samples are required.

The paper is well writen, clear, and the figures are beautiful and interpretable.

Reviewer #3 (Significance (Required)):

This paper presents a VAE approach to generating protein sequences and protein sequence feature extraction. The authors apply the method to predict and model several downstream applications, including protein structure prediction via contact map prediction. Although the method and the paper were interesting, I have some remaining questions about novelty, performance and justification for the architecture used.

In particular I worry that the paper rests on empirical results focused on a few folds/functions and not a systematic test.

As argued in the text we considered the family of luciferase-like proteins a useful testbed for generative model-based design approaches due to the possibility of a simple luminescence-based test for function. We see no reason to expect that similar results could not be obtained with other families of reasonable size. Nonetheless, we agree that it would be helpful to analyse further the importance of the size and constitution of the training dataset, and will add such analysis in the next revision.

I have several critical comments throughout this review, some even suggest big rearrangements, but i am overall positive about the work.

Reviewer #4 (Evidence, reproducibility and clarity (Required)):

**Summary:**

The paper proposed two VAE models to work with aligned sequence input (MSA VAE) and raw sequence input (AR-VAE). It shows the feasibility of using deep generative models to explore the protein sequence space of a family.

**Major comments:**

I believe the authors should qualify some of their claims as preliminary. The study used only one dataset. Some limitations should be discussed. In particular, it is unclear what is found here is general for all protein families. If the authors like to like a high-impact journal, some additional protein families should be studied as well.

We agree that we could do more to discuss the extent to which our results depended on the precise choice of dataset. We hope to go some way towards addressing this concern by retraining models using smaller subsets and performing the same analysis as before, to analyse the extent to which performance depends on the size and precise constitution of the training dataset. We have tried to discuss other limitations in the discussion.

There are some issues with data reproducibility. It is known that VAE (and most other deep learning based methods) depends on how it is trained. Other people using the same method may get different results. It should be pointed out in the paper, although the authors provided the code.

We will seek to better address this in the text. The proposed exploration of the importance of the size and construction of the dataset will provide an additional opportunity to reflect on this aspect of our models and approach, since it requires re-training.

**Minor comments:**

I wouldn't call the cosine similarities between embeddings for pairs of amino acids correlated well based on Fig. 2, which shows the spreading is rather high.

It is true that, especially for substitutions with negative scores, there appear to be deviations. We will revise the claim appropriately.

Reviewer #4 (Significance (Required)):

Multiple studies have been published to generate protein sequence via machine learning. The main part I like for this paper is its thorough assessments of generated sequences in various properties, which have not been done before. Such assessments are valuable.

Compared to other studies, it is not particularly advanced in terms of machine learning. VAE is not as good as GAN and some other models, especially in preserving the sequence properties. If possible, the authors should run GAN on this protein family to see the difference.

We agree that further exploration of different types of model, including GANs, is of interest, but feel that it is beyond the scope of the present work. We are not aware of work that has conclusively established the superiority of GANs for protein sequences, and note that VAEs trained on aligned sequences have shown success in unsupervised fitness effect prediction.

The bioinformatics community would be interested in such a study. My own expertise is bioinformatics.

## References

Bloom J.D., Silberg J.J., Wilke C.O., Drummond D.A., Adami C. & Frances H. Arnold. Thermodynamic prediction of protein neutrality, *Proceedings of the National Academy of Sciences*, 102 (3) 606-611 (2005).

Bowman, S., Vilnis, L., Vinyals, O., Dai, A., Jozefowicz, R. & Bengio, S. Generating sentences from a continuous space, in *Proceedings of The 20th SIGNLL Conference on Computational Natural Language Learning* (2016).

Gómez-Bombarelli R., Wei J.N., Duvenaud D., Hernández-Lobato J.M., Sánchez-Lengeling B., Sheberla D., Aguilera-Iparraguirre J., Hirzel T.D., Adams R.P. & Aspuru-Guzik A. Automatic Chemical Design Using a Data-Driven Continuous Representation of Molecules**,** *ACS Central Science*, 4 (2), 268-276 (2008).

Greener, J.G., Moffat, L. & Jones, D.T. Design of metalloproteins and novel protein folds using variational autoencoders. *Sci Rep* 8, 16189 (2018).

Hou Q., Kwasigroch J.M., Rooman M. & Pucci F. SOLart: a structure-based method to predict protein solubility and aggregation, *Bioinformatics*, Volume 36, Issue 5, Pages 1445–1452 (2020).

Riesselman, A.J., Ingraham, J.B. & Marks, D.S. Deep generative models of genetic variation capture the effects of mutations. *Nat Methods* 15, 816–822 (2018).

Sarkisyan, K., Bolotin, D., Meer, M. *et al.* Local fitness landscape of the green fluorescent protein. *Nature* 533, 397–401 (2016).
