# Supplementary material for: Generating functional protein variants with variational autoencoders
Source: PLoS Comput Biol. 2021 Feb 26;17(2):e1008736. doi: 10.1371/journal.pcbi.1008736 (PMC7946179; doi:10.1371/journal.pcbi.1008736)
Supplement: Attachment — Submitted filename: PLOSCB_revisions_reviewer_responses.docx [file pcbi.1008736.s011.docx]

**Reviewer #1:** The authors addressed reviewers’ comments thoroughly. The revised version is improved in quality. I have no further suggestions to make.

Thank you for the positive evaluation.

**Reviewer #2:** The paper presents a study of generating sequences for luciferase domains based on training VAEs from a set of 70,000 protein sequences similar to luciferase sequences. Some sequences from each model are realised and their activity measured.

The generated sequences mostly do luminesce, though none as strongly as WT. [It would be good to know about more controls than just the one -are any of the 70k sequences better than the measured WT?)

It is important to highlight here that the vast majority of the 70k sequences are not luciferases. The protein family used contains mostly oxidoreductases with a vast range of substrates. In this dataset only ~150 proteins close to luxA (IPR033924) likely reduce FMNH2 and emit light. The luciferase system of *Photorhabdus luminescence* is broadly used in microbiology applications because of its strong luminescence levels. We also note that in our revised submission, we have repeated our measurements of luminescence with a new experimental setup designed to reduce the background noise level, and find that some of the generated sequences do retain WT levels of luminescence. Nonetheless, it remains true that most generated sequences luminesce less strongly than the wild type. Given the dimeric nature of bacterial luciferase, it is possible that interactions between the modified luxA and the WT luxB subunit may contribute to the lower luminescence levels for most generated sequences.

The need to do this (given there are 70,000 examples) is not strongly motivated in itself, but the paper did go on to show conditional versions where solubility of the generated sequences is controlled, while retaining function, though it’s not shown whether this is better or worse than just directly modifying the WT to adjust solubility in ways that are well-known (especially given the extensive MSA - cf PROSS).

Protein engineering approaches such as directed evolution rely on a random search of local sequence space. Generative models able to effectively search sequence space for likely functional variants offer the potential to improve the efficiency of such approaches. Even for large families, the space of unexplored functional protein sequences is likely far larger than that of existing, evolutionarily accessible sequences, and exploring this unexplored sequence space efficiently could be of significant benefit in e.g. library design for high throughput functional screens. A minimum requirement for such benefit to be realised is that generative models should be able to reliably produce functional sequences, which is what we seek to establish here. Some of the other works that we cite in the discussion have already suggested ways in which such generative models could be exploited for optimisation of sequence function (e.g. Brookes and Listgarten, 2019). We see the generative approach as largely complementary to other approaches which exploit other forms of information or predictors, and seek to use the solubility conditioning task as a validation of this approach. In particular, PROSS combines atomistic modelling (requiring a known structure) with constraints derived from the first order statistics of a multiple sequence alignment (similar to what is summarised by a profile HMM). We note that our approach does not require a structure and that our results showing the improved ability of our models to prioritise functional variants relative to the profile HMM indicate that hybrid methods such as PROSS could potentially benefit from incorporating constraints derived by fitting models capable of modelling higher-order interactions to MSAs.

I find that the authors in both the paper and the response to previous reviewers’ comments do seem to consider “edit distance” a bit naively. Yes, 5 random mutations [uniform in position and across AA type] are very likely to destroy function, but it would not be hard to choose sensible mutations that are unlikely to destroy function, especially given such an extensive MSA.

We agree that it is possible to imagine a variety of strategies that do better than random mutation. However, at the kinds of edit distances to a given protein considered here, the combinatorial space of possible mutations is vast, and selection of (potentially large numbers of) functional variants from such a space is non-trivial. For example, as noted in our responses to previous reviewers, evidence from empirical fitness landscapes suggests that not only random mutations, but also sets of mutations selected from orthologues, are likely to destroy function at this range. In the GFP study of Sarkisyan et al. it was found that while “*mutations with a deleterious impact were less likely to be found in orthologous sequences...a substantial fraction of genotypes containing only mutations leading to amino acid states from GFP orthologues was non-fluorescent*”. Indeed, although the vast majority of sequences containing a single substitution to another amino acid observed in an MSA of GFP orthologs tested were found to retain fluorescence, this decreased to 64% (48/75) of sequences containing 6 such mutations, and only 53% (9/17) of those containing 7 such mutations.

Previous work has also demonstrated that simply replicating first order statistics from MSAs does not suffice to recapitulate function (Socolich et al., 2005).

We therefore argue that (i) (generative) approaches which are capable of exploring the functional neighbourhood of a given protein are of considerable interest for protein design, particularly if they do so in an entirely data-driven manner (ii) prior understanding of fitness landscapes suggests that this is a non-trivial task with vastly more non-functional than functional sequences (iii) the comparisons with BLOSUM and HMM-based mutation scoring schemes included in our previous responses to reviewers and in our revised manuscript help differentiate our models from simpler MSA-based scoring schemes.

I agree with the previous reviewers (and the authors) that there is a lot of work in this direction, and this is very limited by needing such a large dataset of similar proteins whose function we're just recapitulating. I do nevertheless find it worthy enough for publication. I find the contrast between the “MSA” and AR versions of the model interesting.

**Reviewer #3:** Overall, the authors adequately addressed the issues raised in the initial review and present a reasonable plan for a re-submission. There are, however, some remaining points that should be addressed:

To demonstrate the value of conditioning on solubility, and support the claim that the conditional model can be used to “control the solubility level while introducing relatively few mutations”, the authors show a plot which compares the predicted solubility of sequences generated by the conditional model and sequences in the training set. To better support for these claims, the authors should also include the predicted solubility of sequences generated by the model without conditioning on solubility. This would more directly address the value of using a conditional model.

The plot shows that it is possible to produce sequences close to P19839 luxA with higher predicted solubility levels at a given distance than any natural sequence at a similar distance. The conditional model is therefore able to transfer an understanding of the properties of soluble sequences in general to enhance the solubility of the non-soluble sequence P19839.

To the extent that an unconditional model successfully captures the true data distribution, it is reasonable to expect variants sampled from the model at a given distance should reflect the distribution of actual solubility levels at that distance. In practice we also find that the level of solubility of variants generated by unconditional models is sensitive to the random seed used to control the initialisation of weights and the stochastic optimisation procedure. We therefore include an additional plot comparing unconditional and conditional models trained across multiple random seeds by the typical levels of solubility reached by the top variants. Although these results indicate that it is also possible to increase the solubility of luxA with an unconditional model, the degree of increase that can be achieved depends on the random seed. Conditioning consistently achieves good results across random seeds, and allows more efficient sampling of diverse high solubility variants.

*Figure: Predicted solubility of top variants generated by conditional and unconditional MSA VAE models across 10 random seeds. 500 variants were sampled from the posterior of the unconditional MSA VAE models and the highest level of the conditional models. For each random seed and each model, we computed the predicted solubility values of all sequences within 30 amino acid differences to luxA P19839. As a measure of the ability of the models to generate diverse high-solubility variants, we show the mean of the 50th highest predicted value across seeds. Error bars represent standard deviations. Three initialisations of the conditional model and three initialisations of the unconditional model generated insufficient variants within the distance threshold and were therefore excluded.*

To address a concern (raised by multiple reviewers) that the model should be more extensively compared to baselines, the authors present some additional analysis, where they show that the model ELBOs show a higher correlation with protein luminescence than BLOSUM or HMM scores. The results shown provide compelling evidence that the MSA VAE can generate novel variants that maintain luciferase activity. The HMM does, however, reasonably predict the luminescence of AR-VAE generated sequences, suggesting that one could potentially generate functional proteins by sampling from the HMM.

We agree that the results indicate that it might indeed be possible to generate variants by, for example, selecting mutations on the basis of their scores under the HMM, while noting that the performance of the HMM scores is worse than those of either VAE model. Such an approach would constitute a variant of “consensus design” (Porebski et al., 2016). The HMM’s inability to capture higher-order interactions, whose importance in stability and function has been established (e.g. Socolich et al., 2005), is an important motivation for developing new generative models for unaligned sequence data.

A final, minor, point is that the authors indicate that the sequences generated using the conditional model are more similar to the wild type sequence than sequences generated by the VAE without conditioning on solubility. Aggregate statistics for their luciferase assays are nonetheless reported in the abstract. This is misleading and is an overestimate of the performance of the non-conditional model. The number of functional variants that are far away from training sequences is less than the presented amount.

We apologise for any confusion generated from aggregating the results. We have now modified the abstract as follows:

The vast expansion of protein sequence databases provides an opportunity for new approaches which seek to learn the sequence-function relationship directly from natural sequence variation. Deep generative models trained on protein sequence data have been shown to learn biologically meaningful representations helpful for a variety of downstream tasks, but their potential for direct use in protein engineering remains largely unexplored. Here we show that variational autoencoders trained on a dataset of almost 70000 luciferase-like oxidoreductases can be used to generate novel, functional variants of the luxA bacterial luciferase. We propose separate VAE models to work with aligned sequence input (MSA VAE) and raw sequence input (AR-VAE), and offer evidence that while both are able to reproduce patterns of amino acid usage characteristic of the family, the MSA VAE is better able to capture long-distance dependencies reflecting the influence of 3D structure. To validate the practical utility of the models, we used them to generate variants of luxA whose luminescence activity was validated experimentally. We further showed that conditional variants of both models could be used to increase the solubility of luxA without disrupting function. Altogether 6/12 of the variants generated using the unconditional AR-VAE and 9/11 generated using the unconditional MSA VAE retained measurable luminescence, together with all 23 of the less distant variants generated by conditional versions of the models; the most distant functional variant contained 35 differences relative to the nearest training set sequence. These results demonstrate the feasibility of using deep generative models to explore the space of possible protein sequences and generate useful variants, providing a method complementary to rational design and directed evolution approaches.

**References**

*David Brookes, Hahnbeom Park, Jennifer Listgarten*, Conditioning by adaptive sampling for robust design; Proceedings of the 36th International Conference on Machine Learning, PMLR 97:773-782, 2019. <http://proceedings.mlr.press/v97/brookes19a.html>

Benjamin T. Porebski, Ashley M. Buckle, Consensus protein design, *Protein Engineering, Design and Selection*, Volume 29, Issue 7, July 2016, Pages 245–251, <https://doi.org/10.1093/protein/gzw015>

Socolich, M., Lockless, S., Russ, W. *et al.* Evolutionary information for specifying a protein fold. *Nature* 437, 512–518 (2005). <https://doi.org/10.1038/nature03991>
